# Immunoglobulin G4-related sclerosing cholecystitis presenting as gallbladder cancer: a case report

**DOI:** 10.1186/s40792-015-0123-4

**Published:** 2015-12-03

**Authors:** Kodai Takahashi, Hideto Ito, Toshio Katsube, Ayaka Tsuboi, Masatoshi Hashimoto, Emi Ota, Kazuhito Mita, Hideki Asakawa, Takashi Hayashi, Keiichi Fujino, Sigeru Okamoto

**Affiliations:** Department of Surgery, New Tokyo Hospital, 1271 Wanagaya, Matsudo City, Chiba Japan; Department of Pathology, New Tokyo Hospital, 1271 Wanagaya, Matsudo City, Chiba Japan

**Keywords:** IgG4-related sclerosing disease, IgG4-related cholecystitis, Gallbladder cancer

## Abstract

Immunoglobulin G4 (IgG4)-related sclerosing disease is a systemic inflammatory syndrome, and an understanding of its characteristics is currently evolving. IgG4-related cholecystitis is a manifestation of IgG4-related sclerosing disease in the gallbladder. This case report describes the clinical, radiographic, and histopathological findings in a young male patient who presented with a synchronous mass in the gallbladder. Serum levels of IgG4 and the IgG4/IgG ratio were normal, and there was no associated autoimmune pancreatitis. Therefore, establishing a preoperative diagnosis of IgG4-related cholecystitis was very difficult, and a differential diagnosis of gallbladder cancer infiltrating the liver was suggested. Postoperative histopathological examination established a diagnosis of IgG4-related cholecystitis definitively. A preoperative diagnosis of IgG4-related cholecystitis, although possible, would have been highly challenging in this case. It is difficult to establish whether surgical intervention is necessary in IgG4-related cholecystitis. Because malignant tumors are frequently suspected with this clinical presentation, surgical intervention should be undertaken only after due deliberation.

## Background

Immunoglobulin G4 (IgG4)-related sclerosing disease, a systemic inflammatory syndrome characterized by obliterative phlebitis and extensive infiltration of IgG4-positive plasma cells and lymphocytes with fibrosis in the pancreas, gallbladder, bile duct, salivary gland, kidney, retroperitoneum, and esophagus [1–5], has been increasingly recognized in the past few years.

IgG4-related cholecystitis does not present with characteristic symptoms of cholecystitis and is often accompanied by IgG4-related sclerosing cholangitis (IgG4-SC). The major clinical symptoms of IgG4-related cholecystitis or IgG4-SC are obstructive jaundice, nausea, fatigue, loss of appetite, and yellowish discoloration of the skin and frequently lead to bile duct stenosis.

IgG4-related sclerosing disease generally displays a preponderance in elderly males, frequent elevation of serum IgG4 levels, and dramatic response to steroid therapy. In most patients with this disease, serum IgG4 levels are elevated but can be normal [6]. A serum IgG4 within the normal range makes it more difficult to diagnose IgG4-related sclerosing disease. Moreover, it is difficult to discriminate a malignancy based on lymphadenopathy and mass-forming lesion. Making a preoperative diagnosis proves challenging, and the disease is frequently misdiagnosed. As malignant tumors are frequently suspected on the basis of radiologic imaging and clinical symptoms, IgG4-related sclerosing disease should be considered in the differential diagnosis to avoid unnecessary surgical intervention. However, in the current clinical scenario, in cases where the serum IgG4 level is within the normal range and a malignancy cannot be ruled out on preoperative diagnostics, surgical intervention becomes necessary.

To date, there have been several reports in the literature of IgG4-SC misdiagnosed as biliary cancer. However, IgG4-related cholecystitis mimicking gallbladder cancer has been rarely reported [7–9]. Here, we report the rare case of an 18-year-old man with IgG4-related cholecystitis mimicking gallbladder cancer and presenting with normal serum IgG4 levels.

## Case presentation

An 18-year-old man was referred to our hospital with acute onset of painless obstructive jaundice, nausea, fatigue, loss of appetite, and icterus but no fever or past history of similar complaints.

### Clinical presentation

Laboratory tests revealed the following results (values in parentheses indicate normal range): white blood cell count 5270/mm^3^ (3500–9700/mm^3^), hemoglobin 14.5 g/dL (13.6–18.3 g/dL), hematocrit 43.2 % (40.4–51.9 %), platelet count 295,000/mm^3^ (140,000–379,000/mm^3^), aspartate aminotransferase 214 IU/L (0–40 IU/L), alanine aminotransferase 314 IU/L (5–45 IU/L), alkaline phosphatase 2367 IU/L (104–338 IU/L), total bilirubin 8.1 mg/dL (0.3–1.2 mg/dL), direct bilirubin 6.0 mg/dL (0–0.4 mg/dL), amylase 74 U/L (39–134 U/L), total protein 8.1 g/dL (6.5–8.2 g/dL), albumin 4.4 g/dL (3.7–5.5 g/dL), and urine bilirubin 3+ (negative). Serum carcinoembryonic antigen (CEA) and carbohydrate antigen 19-9 (CA 19-9) levels were within normal limits as was the serum IgG4 (40 mg/dL; range, 4–108 mg/dL) and IgG (1055 mg/dL; range, 820–1740 mg/dL) levels.

**Fig. 1:**
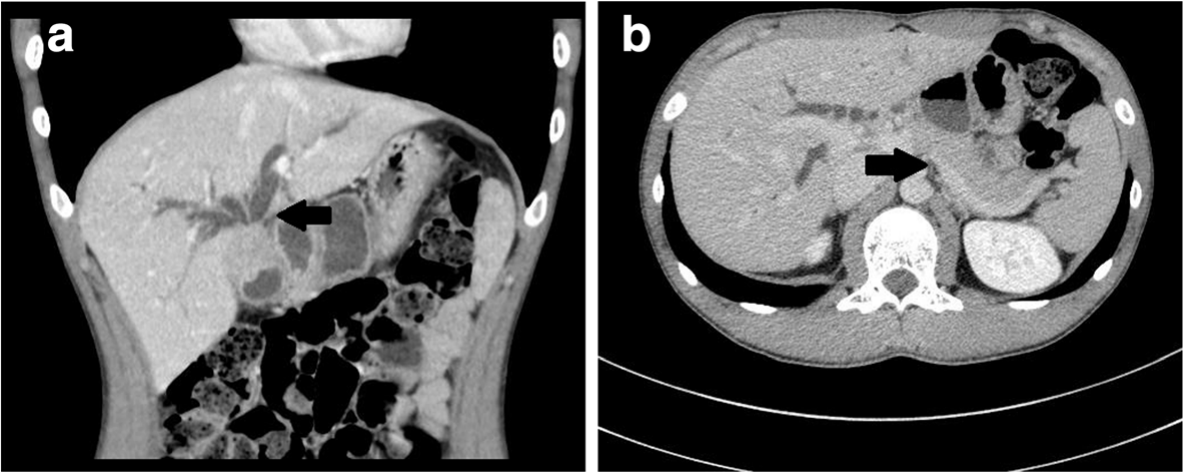
Pathologic findings on CT. **a** CT shows abnormal thickening of the gallbladder wall that appeared to invade the adjacent portion of the liver. An infiltrative low-density mass (30 mm) involving the gallbladder neck, upper biliary tract, and hilar bile duct as well as intrahepatic bile duct dilatation are visible (annotated with a *black solid arrow*). **b** The pancreas was not enlarged (annotated with a *black solid arrow*)

**Fig. 2:**
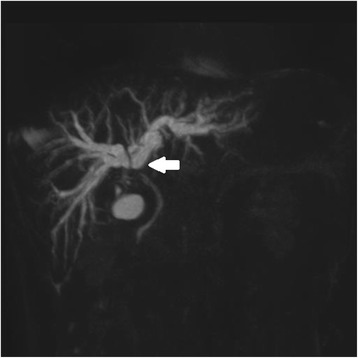
Infiltrative lesion on MRCP. MRCP shows an infiltrative mass involving the gallbladder neck, upper biliary tract, and hilar bile duct with high-signal intensity on diffusion-weighted image. In addition, a sharp-beaked stenosis of the hilar bile duct with upstream bile duct dilatation was noted (annotated with a *white solid arrow*)

**Fig. 3:**
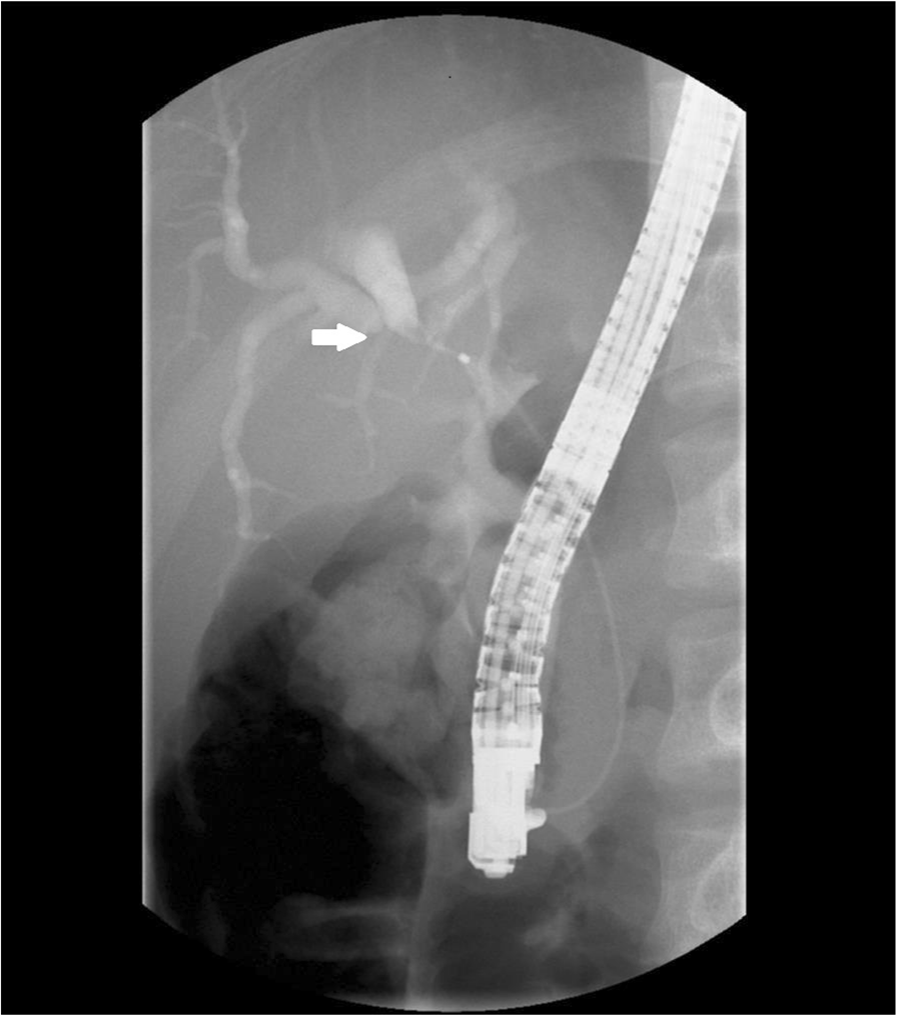
Postinterventional ERCP. ERCP shows that both the intrahepatic bile ducts were dilated and a plastic stent was placed (annotated with a *white solid arrow*)

Contrast-enhanced computed tomography (CT) revealed abnormal thickening of the gallbladder wall, which appeared to invade the adjacent portion of the liver. Other imaging findings included an infiltrative low-density mass (30 mm; with mild enhancement in the arterial and delayed phases) involving the gallbladder neck, upper biliary tract, and hilar bile duct as well as intrahepatic bile duct dilatation. The pancreas was not enlarged (Fig. 1a, b). Magnetic resonance imaging (MRI) and magnetic resonance cholangiopancreatography (MRCP) revealed an infiltrative mass involving the gallbladder neck, upper biliary tract, and hilar bile duct with high-signal intensity on diffusion-weighted imaging. Furthermore, there was a sharp-beaked stenosis with an upstream dilatation of the hilar bile duct. The main pancreatic duct appeared normal without any malfusion of pancreaticobiliary ducts (Fig. 2). Endoscopic retrograde cholangiopancreatography (ERCP) revealed a bile duct stricture similar to that observed on MRI; for this, both the intrahepatic bile ducts were dilated and a plastic stent was placed (Fig. 3). An endoscopic ultrasound revealed a heterogeneous hypoechoic tumor measuring 9.3 × 7.3 mm^2^. Doppler ultrasonography of the abdomen revealed a hypoechoic lesion of the gallbladder that showed good vascularity and direct hepatic invasion as well as intrahepatic bile duct dilatation. Exfoliative cytology of the bile duct revealed inflammatory cells without atypia. Positron emission tomography (PET)-CT was not performed. As a malignancy could not be ruled out, surgical resection was proposed. First, we decided to perform right hemihepatectomy with caudate lobectomy. However, we chose extended cholecystectomy and intrahepatic cholangiojejunostomy because we found a benign region for intraoperative consultation. We had to resect the common bile duct because the inflammation of the gallbladder was severe and had spread to the common bile duct.

**Fig. 4:**
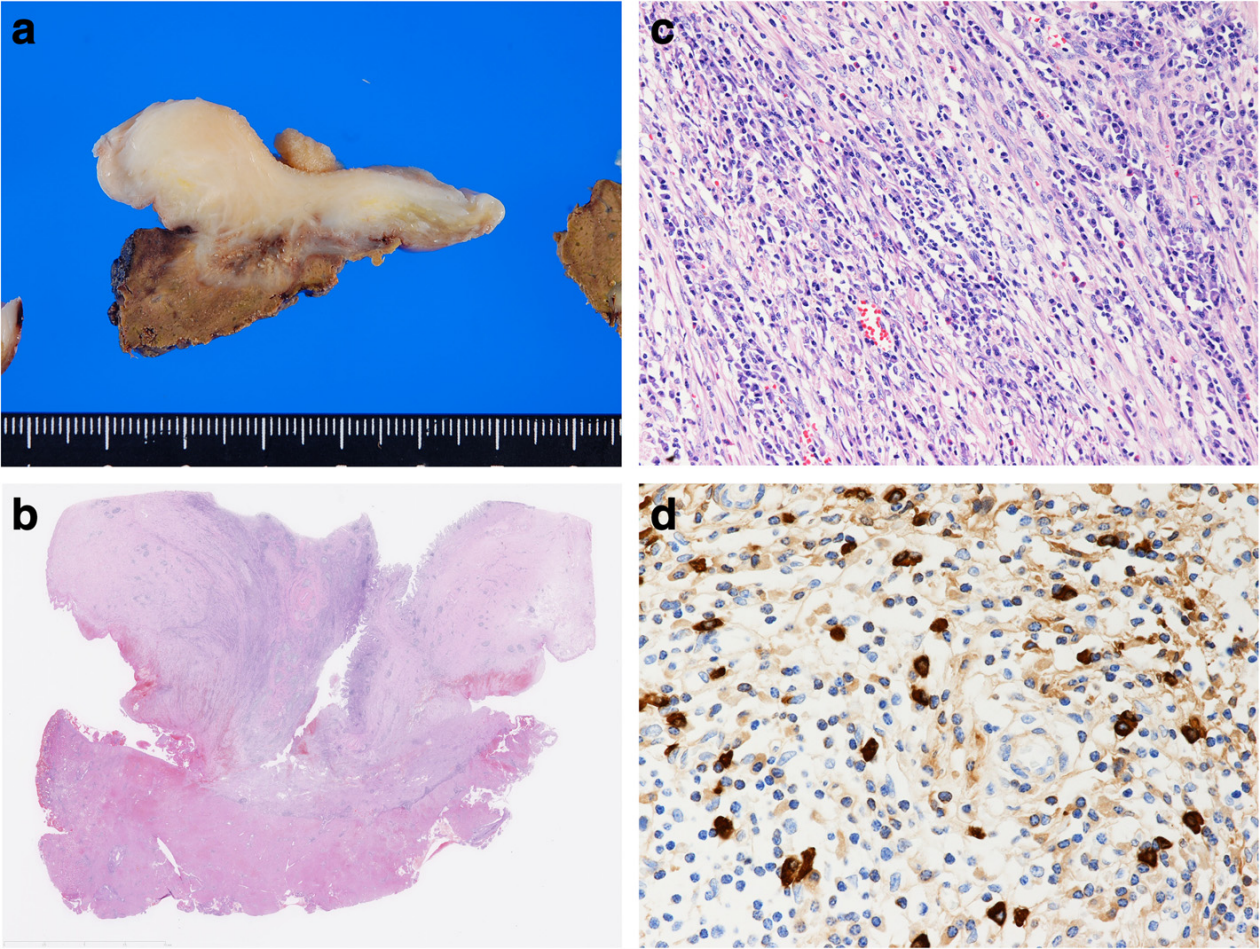
**a** Gross findings revealed a 4.5-cm mass involving the gallbladder and liver hilum. **b** Microscopic findings revealed diffuse lymphoplasmacytic infiltration, dense fibrosis, and obliterative phlebitis. **c** Other findings revealed infiltration of some eosinophils and neutrophils and some xanthogranulomatous inflammations. **d** Immunohistochemical staining for IgG4 showed IgG4/plasma cell 10–40 % and many IgG4-positive plasma cells (maximum density 50 per high-power field)

Gross pathologic examination of the surgical specimen (a 4.5-cm mass) revealed invasion of the gallbladder and hilum of the liver; the liver was depressed by the mass but not invaded (Fig. 4a). Histopathological examination revealed diffuse lymphoplasmacytic infiltration and dense fibrosis and a diffuse fibrohistiocytic inflammatory pseudotumor of the gallbladder (Fig. 4b). Obliterative phlebitis, eosinophilic and neutrophilic infiltration, and some xanthogranulomatous inflammation were also observed (Fig. 4c). No histologic signs of malignancy were detected; however, storiform fibrosis and lymphoplasmacytic infiltration were present. Immunohistochemical staining for IgG4 showed the IgG4/plasma cell ratio was 10–40 % and many IgG4-positive plasma cells (maximum density, 50 per high-power field) (Fig. 4d). The mucous membrane of the common bile duct was normal. As the histological findings were highly suggestive of IgG4-related disease, the patient was diagnosed with IgG4-related cholecystitis.

The postoperative course was uneventful, there was no added steroid therapy, and the patient was discharged 17 days after the surgery without any additional treatment. At the 1-year follow-up, the patient had no symptoms or signs of the disease.

### Discussion

In recent years, IgG4-related sclerosing disease has become an increasingly intriguing clinicopathologic entity. The pathology involves diffuse or focal organ enlargement and mass-forming or thickening lesions, caused by heavy infiltration of IgG4-positive plasma cells and lymphocytes with fibrosis [10–12], in various organs, with resultant autoimmune pancreatitis, sclerosing cholangitis, cholecystitis, sialadenitis, retroperitoneal fibrosis, inflammatory aortic aneurysm, or inflammatory pseudotumors. However, the exact pathogenesis and pathophysiology of IgG4-related sclerosing disease remains unclear. As a malignancy is frequently suspected on the basis of clinical symptoms and radiologic findings in such cases, IgG4-related sclerosing disease should be included as a differential diagnosis. In the present case, diseases contributing to stenosis of the bile duct included cholangiocarcinoma, infiltration of the bile duct by gallbladder cancer, PSC, IgG4-related sclerosing disease, ischemic bile duct stenosis, and an amputation neuroma. In most cases, the diagnosis is easily established; however, a preoperative diagnosis is difficult to establish in a few patients.

Diagnostic criteria for IgG4-related sclerosing disease were published by Deshpande et al. [13] wherein the three major histopathological features reported to be associated with IgG4-related sclerosing disease are dense lymphoplasmacytic infiltration and fibrosis, arranged at least focally in a storiform pattern, and obliterative phlebitis. Other histopathological features associated with IgG4-related sclerosing disease include phlebitis without obliteration of the lumen and eosinophilia. Minimal criteria for IgG4-related sclerosing disease in a new organ/site are characteristic histopathological findings with elevated IgG4 plasma cell numbers, increased IgG4/IgG ratio that is considered characteristic [6], high serum IgG4 levels, quick response to steroid therapy, and reports of multiorgan involvement consistent with IgG4-related sclerosing disease (Table 1). On histopathology of surgical specimen, 50 or more IgG4 plasma cells per high-power field and an IgG4/IgG plasma cell ratio greater than 40 % are highly suggestive of IgG4-related sclerosing disease [14].

**Table 1:** Three major histopathological features and international pathological consensus minimal criteria for diagnosing IgG4-related disease in a new organ/site

The three major histopathological features associated with IgG4-related disease
①Dense lymphoplasmacytic infiltrate
②Fibrosis, arranged at least focally in a storiform pattern
③Obliterative phlebitis
Other histopathological features associated with IgG4-related disease are as follows:
①Phlebitis without obliteration of the lumen
②Eosinophilia
Minimal criteria for IgG4-related disease in a new organ/site
①Characteristic histopathological findings with an elevated IgG4 plasma cells and IgG4/IgG ratio
②High serum IgG4 concentrations
③Effective response to glucocorticoid therapy
④Reports of other organ involvement that is consistent with IgG4-related disease

Most IgG4-related sclerosing diseases are associated with autoimmune pancreatitis (AIP) [15], which was not the case in our patient. This case was unique in that the patient was considerably younger and had IgG4-related cholecystitis mimicking gallbladder cancer with a normal IgG4 concentration. Normal serum IgG4 levels in IgG4-related sclerosing disease have been reported only in a few articles [7, 9, 16], and the sensitivity and specificity of serum IgG4 for IgG4-related SC is reported to be 50 and 60 %, respectively [17]. In cases with suspected IgG4-related cholecystitis, other benign gallbladder diseases such as xanthogranulomatous cholecystitis etc., gallbladder cancer should be a differential diagnosis. Therefore, serum IgG4 should be assessed. However, serum IgG4 and the IgG4/IgG ratio were normal in this case, and without the associated AIP, a preoperative diagnosis was very difficult and a histopathological diagnosis was necessary for a definitive diagnosis. Bile cytology indicated no malignancy in this patient. However, bile cytology is often normal in cholangiocarcinoma. We tried endoscopic ultrasonography fine-needle aspiration (EUS-FNA). However, we did not succeed. EUS-FNA was useful for diagnosing IgG4-related sclerosing disease. In AIP, EUS-FNA provided tissue samples adequate for histopathological evaluation and greatly contributed to the histological diagnosis [18]. We did not perform other pathological examinations because the patient was developing worse obstructive jaundice rapidly in a short time. In general, other diagnostic approaches include biopsies of the gallbladder, bile duct, and liver. A previous study suggested that biopsies of the gallbladder, bile duct, and liver are useful for diagnosing IgG4-related cholecystitis [19–22]. Biopsies of the bile duct and gallbladder, although technically difficult, could provide the full spectrum of morphologic changes. In general, fibroinflammatory involvement is mainly observed in the submucosa of the gallbladder and bile duct wall, whereas the epithelium of these structures was intact in the present case. Cytological examinations were often useful in establishing a differential diagnosis of gallbladder cancer, although it was difficult to obtain biopsy samples adequate for establishing characteristic histopathological findings of IgG4-related cholecystitis [22]. Liver biopsies have the lowest diagnostic potential, although sometimes useful in the diagnosis of IgG4-related cholecystitis in cases of intrahepatic bile duct involvement [22]. Therefore, we thought EUS-FNA or biopsy was useful for a differential diagnosis between gallbladder cancer and IgG4-related cholecystitis. We did not conduct other pathologic examinations in the present case because of the technical difficulty involved in performing a biopsy as well as the inadequacy of the tumor mass required for a biopsy. The quality of biopsy samples depends on the experience of the endoscopists undertaking the procedure; moreover, biopsy samples were sometimes small or had artificial degeneration. We did not perform PET in the present case as a report of PET that revealed an elevated standardized uptake value, associated with malignant tumor, has been published previously [16] and was not considered beneficial to establish a differential diagnosis.

In view of all of these aspects, establishing a preoperative diagnosis of IgG4-related cholecystitis was possible but highly challenging and it was too difficult to determine whether surgical intervention was actually necessary in this case. This patient was developing worse obstructive jaundice rapidly in a short time. We did not perform both PET and EUS-FNA. In this case, a preoperative diagnosis was difficult. We could not assess the benign region (xanthogranulomatous cholecystitis or IgG4-related cholecystitis) or cancer; therefore, we decided to perform operation and intraoperative consultation. Although there is much to be understood with regard to IgG4-related cholecystitis, it is conceivable that a trial of steroid therapy may be warranted if this disease entity is suspected before surgery. A poor response to steroid therapy should indicate the possibility of a diagnosis of gallbladder cancer with a need for re-evaluating the diagnosis. Thus far, there are several reasons why a definitive preoperative diagnosis cannot be established in such a case. Moreover, because malignant tumors were frequently suspected under these conditions, surgical intervention should be undertaken after due deliberation.

At present in Japan, there are no reports describing a presentation of IgG4-related cholecystitis without AIP. Herein, we report the first case in the literature of a young man with only IgG4-related cholecystitis and normal serum IgG4 concentrations.

In 2010, Leise et al. reported a case of an ill-defined tumor (6 cm) in the liver hilum that appeared to surround the gallbladder, which was established to be IgG4-related cholecystitis on examination of the surgical specimen [23]. In 2005, Gumbs et al. reported the appearance of synchronous tumors in the pancreatic head and gallbladder that were preoperatively diagnosed as pancreatic ductal adenocarcinoma and gallbladder cancer [24]. Postoperative histopathological examination established AIP with IgG4-related cholecystitis. Some other cases reported IgG4-related cholecystitis mimicking gallbladder cancer [7–9, 23, 24] (Table 2). A few cases of synchronous autoimmune pancreatitis and pancreatic ductal adenocarcinoma have also been reported [25, 26]. In addition, one report describes epithelial atypia in the common bile duct in the presence of bile duct involvement in autoimmune pancreatocholangitis [27]. Previous reports have reported IgG4-SC occurring with cholangiocarcinoma, but no reports of IgG4-related cholecystitis with gallbladder cancer exist so far. We intend to continue the follow-up of the present case in the outpatient setting.

### Conclusions

In conclusion, we reported the case of a young man with only IgG4-related cholecystitis and normal serum IgG4 concentrations. It was important for preoperative differential diagnosis of IgG4-related cholecystitis from other benign diseases and gallbladder cancer. When we could not diagnose IgG4-related cholecystitis by usual examination, we performed additional examinations. Although we did not succeed, we concluded that EUS-FNA was useful for differential diagnosis. Nevertheless, preoperative diagnosis may be difficult. In that case, operation and intraoperative consultation should be considered.

**Table 2:** Previously reported cases of IgG4-related cholecystitis

Case	Year	Author	Age	Sex	Country	Operation	Surgical form	Associate AIP
1	2005	Gumbs AA[24]	68	M	USA	Yes	Hepatopancreatoduodenectomy	Yes
2	2011	Leise MD[23]	76	M	USA	Yes	Laparoscopic cholecystectomy	Yes
3	2013	Shin SW[7]	58	M	Korea	Yes	Extended cholecystectomy	Yes
4	2013	Lee YS[8]	59	M	Korea	No		Unknown
5	2014	Feely MM[9]	61	F	USA	Yes	Right trisegmentectomy	Unknown
6	2014	Feely MM[9]	71	F	USA	Yes	Extended cholecystectomy	Unknown
7	2014	Feely MM[9]	53	M	USA	Yes	Extended cholecystectomy	Unknown
8	2015	Takahashi K	18	M	Japan	Yes	Extended cholecystectomy	No

## Consent

Written informed consent was obtained from the patient for publication of this case report and accompanying images. A copy of the written consent is available for review by the Editor-in-Chief of this journal.
